# Retraction: Hypoxia-inducible factor 1a induces phenotype switch of human aortic vascular smooth muscle cell through PI3K/AKT/AEG-1 signaling

**DOI:** 10.18632/oncotarget.28575

**Published:** 2024-06-20

**Authors:** Kai Liu, Changcun Fang, Yuwen Shen, Zhengqin Liu, Min Zhang, Bingbing Ma, Xinyan Pang

**Affiliations:** ^1^Department of Cardiovascular Surgery, Qilu Hospital of Shandong University, Jinan 250012, Shandong, China

**This article has been retracted:** Multiple instances of image duplication were discovered in this paper. Specifically, the GAPDH blots in 1st and 2nd panels of Figure 1A are internal duplicates even though the expression of HIF-1α mRNA was measured at different conditions. Additionally, multiple blots in Figure 3A and Figure 8, panels A and B, are duplicates of those in Figure 4B and 4C of an earlier published paper unrelated by subject [1]. Therefore, with the agreement of all authors, the Scientific Integrity office at Oncotarget has decided to retract this paper.

Original article: Oncotarget. 2017; 8:33343–33352. 33343-33352. https://doi.org/10.18632/oncotarget.16448
